# The neural representation of stereotype content

**DOI:** 10.1038/s41598-024-67111-9

**Published:** 2024-07-15

**Authors:** Thérèse Collins, Emilie Zhu, Patrick Rateau

**Affiliations:** 1 Integrative Neuroscience and Cognition Center (UMR 8002), CNRS, Université Paris Cité, 75006 Paris, France; 2 Laboratoire Epsylon (EA 4556), Université Paul-Valéry Montpellier 3, Montpellier, France

**Keywords:** Social stereotype, Stereotype content model, Serial dependence, EEG, Representational similarity analysis, Perception, Human behaviour, Social behaviour

## Abstract

Judgments about social groups are characterized by their position in a representational space defined by two axes, warmth and competence. We examined serial dependence (SD) in evaluations of warmth and competence while measuring participants’ electroencephalographic (EEG) activity, as a means to address the independence between these two psychological axes. SD is the attraction of perceptual reports towards things seen in the recent past and has recently been intensely investigated in vision. SD occurs at multiple levels of visual processing, from basic features to meaningful objects. The current study aims to (1) measure whether SD occurs between non-visual objects, in particular social groups and (2) uncover the neural correlates of social group evaluation and SD using EEG. Participants’ judgments about social groups such as “nurses” or “accountants” were serially dependent, but only when the two successive groups were close in representational space. The pattern of results argues in favor of a non-separability between the two axes, because groups nearby on one dimension but far on the other were not subject to SD, even though that other dimension was irrelevant to the task at hand. Using representational similarity analysis, we found a brain signature that differentiated social groups as a function of their position in the representational space. Our results thus argue that SD may be a ubiquitous cognitive phenomenon, that social evaluations are serially dependent, and that reproducible neural signatures of social evaluations can be uncovered.

## Introduction

When we perceive a person or a group, we rapidly form a judgment that allows us to effectively anticipate real or imagined future interactions^1^. The Stereotype Content Model (SCM) proposes that social relations are represented in a space made of two dimensions characterizing people’s views on a psychological level (^2–4^, for reviews^5,6^). The first dimension, *warmth*, refers to the fact that people and groups cooperate or compete with each other. The assumed intentions of a social target (person or group) translate into judgments of warmth: do the members of this group mean well; does this person harbor positive goals? The second dimension, *competence*, refers to the fact that people and groups differ in the extent to which they possess status, power, and resources. The status, power, and resources believed to characterize a target translate into judgments of competence: does this person or group have the necessary means to reach its goals? These two unmistakable features of social interactions constrain the way perceivers form impressions of groups and group members. In turn, these impressions shape people’s emotional reactions and orient their social behavior^7^.

The value of a social target on the warmth and competence axes defines its location in a two-dimensional representational space which can be seen as characterizing the content of the stereotype. The first goal of the present paper was to examine whether we can discover a neural representation that maps onto this psychological space.

The second goal of this paper was to examine the relationship between warmth and competence. Previous research on this question appears contradictory at first sight. Some work suggests a positive relationship between the two dimensions: the more a social target is judged to be warm, the more it will also be judged as competent^8^. This is also called the halo effect^9^. Although such an effect has often been reported, it concerns almost exclusively the perception of individuals and not that of social groups^10,11^.

Other research suggests that warmth and competence dimensions are orthogonal, such that some social targets may be judged both warm and competent, others warm but incompetent, and so forth. In the area of intergroup relations, the term stereotype has often implied uniform antipathy towards a social group^12–14^. In this view, social judgments are unidimensional, falling along a single good-bad continuum. In contrast, SCM proposes that the two dimensions are orthogonal. Indeed, intentions (the warmth dimension) and the resources that a person is likely to mobilize to achieve them (the competence dimension) can be seen as two independent properties^15^. An individual's intention to achieve a particular goal says nothing about their ability to do so, and conversely, their ability to achieve something says nothing about their intention to do so^4^. Numerous studies on the perception of social groups have supported this postulate of orthogonality between warmth and competence^7,16, 17^.

SCM researchers note that a substantial number of groups tend to be perceived in so-called 'ambivalent' quadrants of the representational space: high competence/low warmth and low competence/high warmth. Glick and Fiske^18,19^, for example, showed that there are two very different stereotypes about women. On the one hand, the traditional, warm, and caring woman who suffers from a perceived lack of competence. On the other, competent professional women and feminists suffer from a perceived lack of warmth. These two stereotypes suggest that beliefs about women are organized around two negatively correlated dimensions.

Similar “mixed stereotypes” were reported by Cuddy and colleagues when they examined the perception of the elderly^20^: when an elderly target was presented as more competent than expected (i.e., an older person who has a sharp memory), participants rated this target as more competent but also as less warm than an elderly target with memory loss. The same applied to the perception of career women^21^. They presented one of two curricula vitae to their participants. The target was a young working woman: in one condition she had no children, whereas in the other condition she was a mother. Results showed that, compared to the childless working woman, the working mother was perceived as both warmer and less competent. What seems to happen is that participants’ stereotypic perception moves from one mixed stereotype (competent and cold) to another (warm and incompetent).

In summary, the orthogonality of the core dimensions of warmth and competence may apply as a general rule, but a negative relationship tends to emerge between the two dimensions when focusing on the perceptions of particular pairs of groups in the context of actual social interactions. In this sense, Yzerbyt, Provost and Corneille^22^ proposed that the two fundamental dimensions of the social perception of groups may in fact be negatively related to each other^23^. More specifically, Yzerbyt and colleagues argued that in the context of two-group comparisons, perceiving group A as superior on one of the two dimensions (e.g. competence) should result in group B being perceived as superior on the other dimension (e.g. warmth). This compensation effect^24^; for a review^25^ appears mainly in situations of intergroup comparison^26^.

The evidence regarding the relationship between warmth and competence can thus be described as inconsistent. One potential reason for this may be that there is, in fact, no systematic relationship. Alternatively, it may be that warmth and competence are systematically related across time and situations, but not in a linear fashion. Imhoff and Koch^27^ suggest that the relationship between the two dimensions is curvilinear: average competence is associated with high warmth, while both low and high competence are associated with low warmth. Ultimately, this means that social groups cannot be perceived as both highly competent and very warm.

The second aim of our study is to contribute to this debate by examining serial dependence between successive social judgments. In other words, does serial dependence provide evidence of psychological separability between warmth and competence dimensions or, on the contrary, of a psychological link between the dimensions of warmth and competence with respect to social groups?

Serial dependence (SD) is a phenomenon known primarily in visual psychophysics: visual objects are perceived to be more like previously seen objects than they really are. Initial studies of SD used simple visual features^28^: when reporting the orientation of a brief Gabor patch, observers tended to err in the direction of the previously seen Gabor. SD also occurs on more complex objects such facial emotional expression^29^ and identity^30^: when reporting the emotional expression of a face (happiness or disgust), or its identity (person A or person B), observers tend to err in the direction of the previously seen face. Serial dependence occurs on perceived facial attractiveness^31^, aesthetic appreciation of art^32^, the direction of a cloud of moving dots^33^, object shape^29,34^, and several other visual objects (for reviews:^35,36^). This diversity of objects on which serial dependence operates suggests that this may be a fundamental aspect of the way we perceive objects: the content of current perception depends, of course, on the current stimulus, but may also integrate information perceived a few seconds in the past.

There are three basic characteristics of serial dependence. First, it is temporally tuned: only sensory information seen in the past few seconds can bias observers’ responses. Second, it is spatially tuned: integration only occurs between objects that occupy nearby locations in space. Finally, it is feature tuned. Integration is maximal between nearby values of a feature (for example, orientations that are less than ~ 30° apart) and drops for larger feature value differences.

This last characteristic is central to our study. If serial dependence occurs in the perception of social groups, then it should manifest between social groups that are nearby in the feature space defined by warmth and competence axes, and not—or less so—between social groups that are far in that feature space. We can thus examine exactly what “near” and “far” mean in this space and draw conclusions about whether and how warmth and competence dimensions interact.

**Figure 1:**
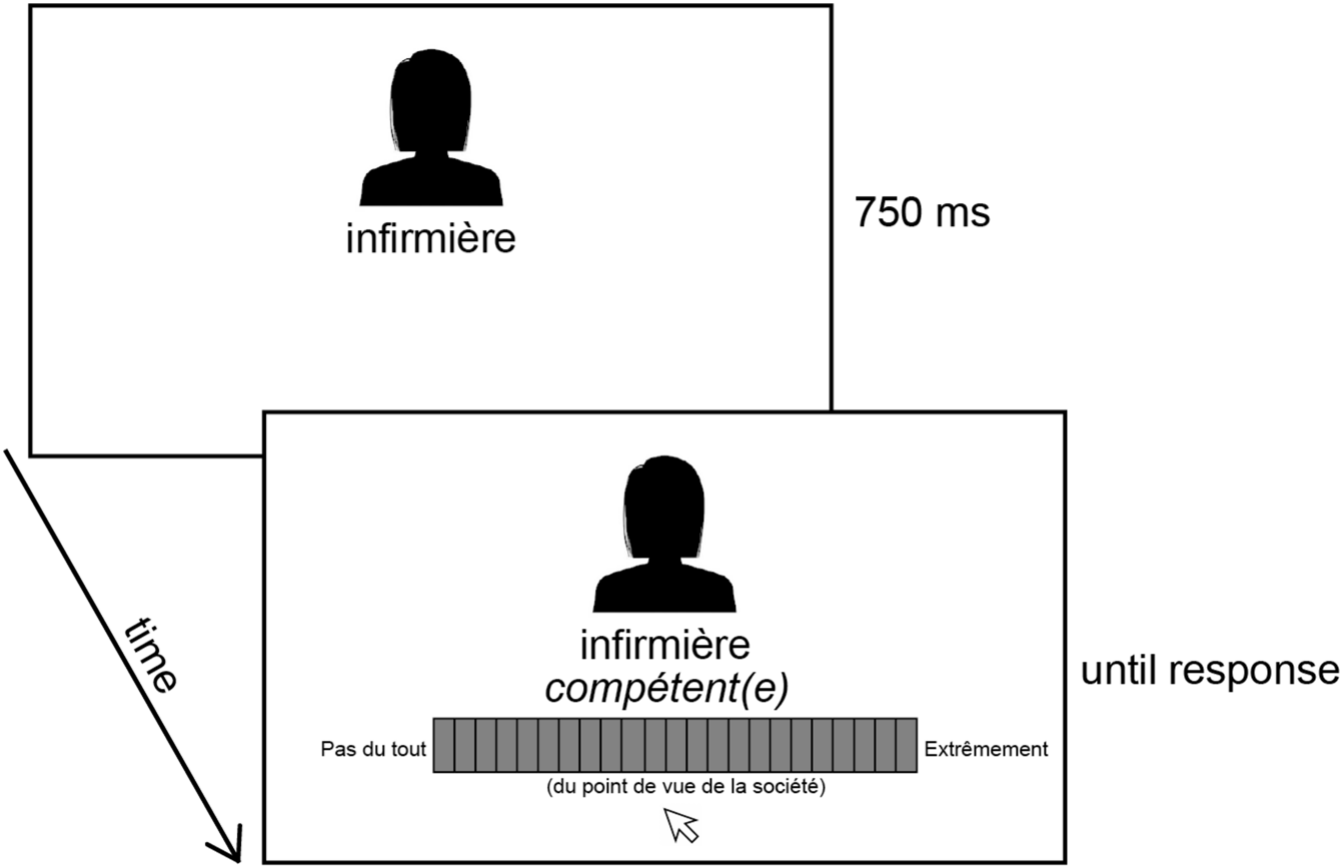
Procedure. A silhouette and social group name, such as “nurse” (*infirmière*) were presented for 750 ms. The response screen was composed of an adjective, such as “competent” (*compétent*(*e*)) and a response bar flanked by “not at all” (*pas du tout*), “very much” (*extrêmement*) and “according to society” (*du point de vue de la société*). The mouse pointer always appeared below the center of the response bar. Participants moved the pointer to click on the level of perceived competence (or whichever of the 8 possible adjectives was present on the screen—see “Methods”).

We asked 36 healthy adult observers to evaluate social groups on warmth and competence scales. While they viewed a social group name (Fig. 1), we recorded the EEG response.

## Results

### Warmth and competence ratings depend on previously seen groups

Participants evaluated social groups on warmth or competence (Fig. 1) in a rapid succession of trials. Each social group belonged to a cluster corresponding to one of the four corners of the space represented by warmth and competence axes (see “Methods”).

**Figure 2:**
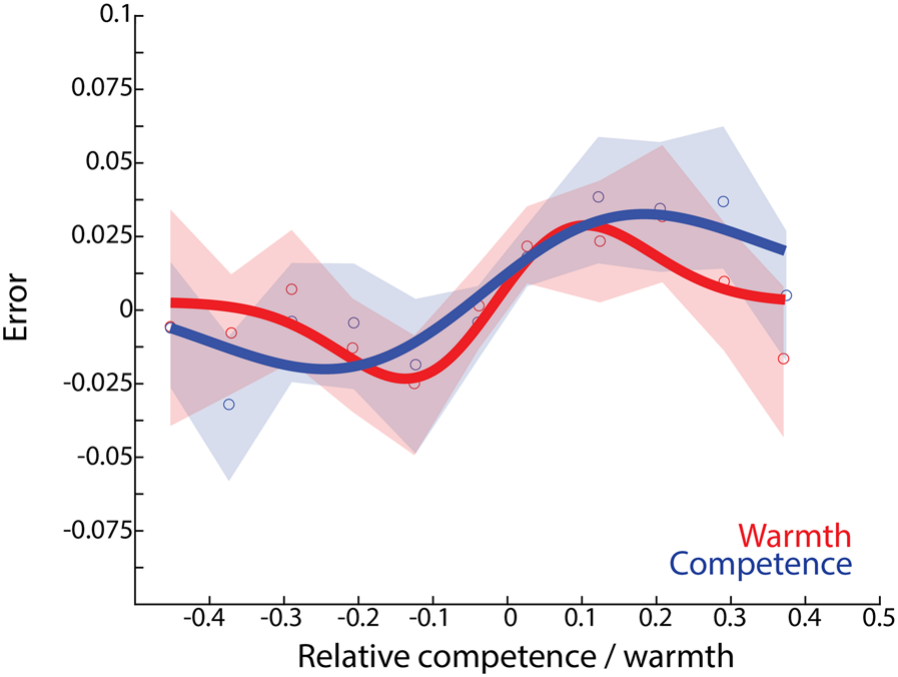
Response error as a function of relative competence (blue) or warmth (red) between successive trials. Data points and shaded 95% confidence intervals are shown, curves show the first derivative of Gaussian fits.

Warmth and competence ratings were serially dependent. Our first measure of SD looked at response error (difference between the response on one trial and the average evaluation of that group across the entire experiment) as a function of relative distance between successive trials. We fit a first derivative of Gaussian (DoG) (see Methods). Figure 2 shows the classic first derivative-of-Gaussian pattern which shows that responses are attracted towards the stimulus seen on the previous trial, but only when that stimulus is nearby in feature space. For both competence and warmth ratings, the $\alpha$ and w parameters were significant (p < 0.05).

**Figure 3:**
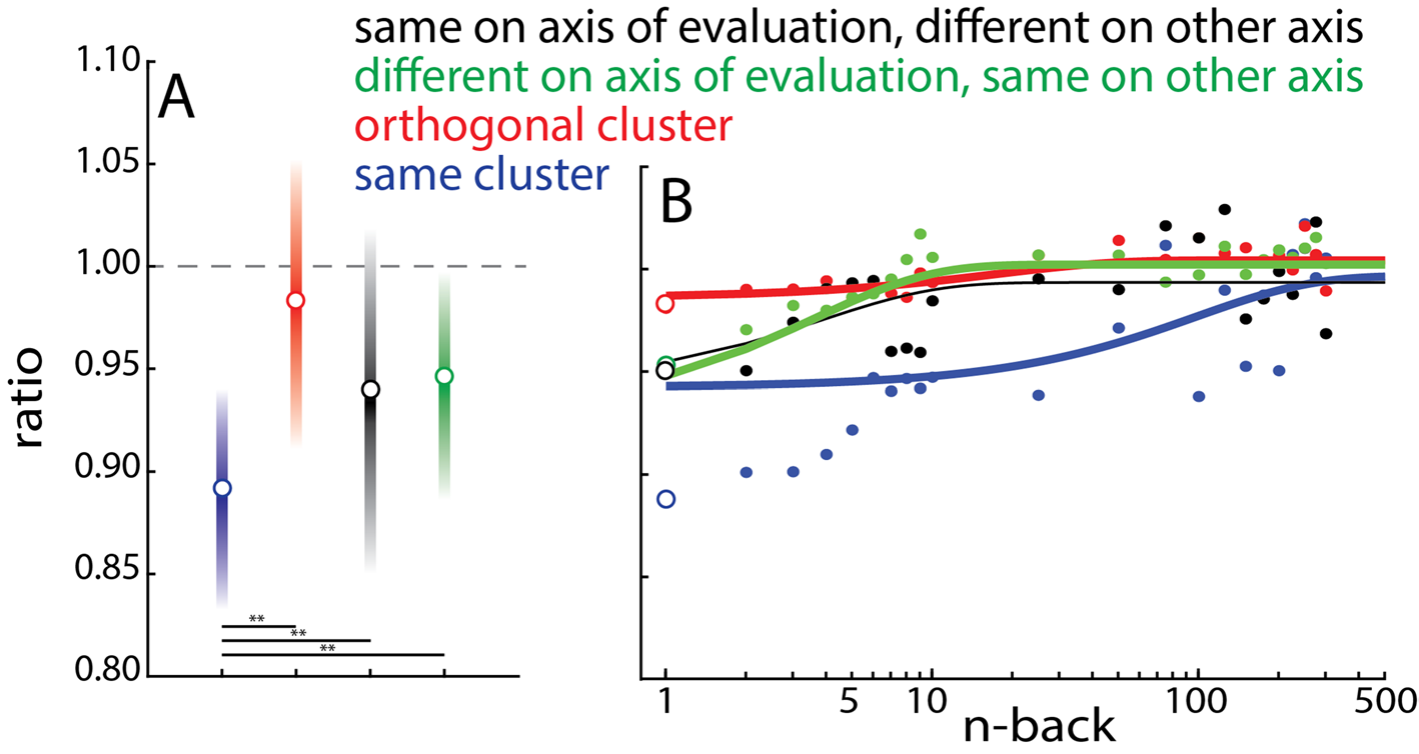
(**A**) Ratio between successive and non-successive trials (1-back) from the same cluster (blue), from orthogonal clusters (red), from clusters nearby on the axis being rated (black) and from clusters far on the axis being rated (green). **p < 0.0001. (**B**) Ratio between successive trials and trials n back in the past for the four conditions.

For the second measure of serial dependence, we looked at the distance between the scores given to two groups, as a function of whether they were in the same or different clusters. We hypothesized that the distance between successive trials should be smaller than the distance between non-successive trials, but only when the two trials were in the same cluster, owing to the feature selectivity of SD. We thus calculated a ratio between successive and non-successive trials, for four types of trial pairs, and for each of the warmth and competence ratings. The first two trial pair conditions were trials from the same cluster and trials from orthogonal clusters. When warmth was being rated, the third condition was trials from clusters with the same warmth but different competence. When competence was being rated, the third condition was trials from clusters with the same competence but different warmth. We then averaged across warmth and competence ratings, and this condition is thus called “nearby on axis being rated, far on other axis” in Fig. 3. The fourth condition, called “far on axis being rated, nearby on other axis” in Fig. 3, averaged over trials from clusters with different warmth but same competence (when warmth was being rated on trial n) and trials from clusters with different competence but same warmth (when competence was being rated on trial n). The ratio for the “mixed” trial pairs is a direct test of whether the warmth and competence axes are psychologically separable. If the axes are separable, then the “nearby on axis being rated, far on other axis” condition should be comparable to the within-cluster condition (ratio less than 1, because successive trials will be rated as more similar than non-successive trials). The “far on axis being rated, nearby on other axis” condition should be comparable to the orthogonal-cluster condition (ratio equal to 1, because the distance in ratings between successive and non-successive trials should be comparable). In other words, if the axes are independent, then the status of a social group on one axis should not interfere at all with its rating on the other. Alternatively, if the axes are not separable, then the third and fourth conditions should present a ratio close to 1. This would mean that social groups of similar warmth and different competence are not as similar to each other as groups of similar warmth and competence (and vice versa).

Figure 3A shows the results, which are compatible with the psychological non-separability of the two dimensions. Indeed, the average ratio in the same-cluster condition was 0.87 [bootstrapped 95% confidence intervals, 0.82–0.92]; in the orthogonal-cluster condition 0.97 [0.91–1.03]; in the “nearby on axis being rated, far on other axis” 0.94 [0.86–1.01]; and in the “far on axis being rated, nearby on other axis”, 0.95 [0.88–1.00]. A repeated-measures ANOVA was run with rating (warmth versus competence) and condition (the four illustrated in Fig. 3 and described above) as factors. Because we had signed hypotheses, the reference alpha level is 0.025. Only the effect of condition was significant (F(1,3) = 35.73, p < 2^–15^). Planned t-tests Bonferroni-corrected for multiple comparisons confirmed a significant difference between same- and orthogonal-cluster conditions [t(35) = 3.3, p < 0.0025, Cohen’s d = 0.21], as well as between same-cluster and “nearby on axis being rated, far on other axis” [t(35) = 3.07, p < 0.005, Cohen’s d = 0.15] and between same-cluster and “far on axis being rated, nearby on other axis” [t(35) = 4.01, p < 0.00005, Cohen’s d = 0.15], but not between orthogonal-cluster and either of the two mixed conditions (p > 0.75).

The SD ratio can be calculated for the 1-back trial (as in Fig. 3A), and for any trial in the past (Fig. 3B). If SD exists, we expect it to decrease with time. In other words, if SD manifests as a ratio of less than one, we expect this ratio to return to one over time. For ease of viewing, trials in the past are presented on a log scale. To characterize the time course, we fit an exponential function to each condition; the resulting fits are represented in Fig. 3B: $ratio = \alpha *exp(\beta *nback)+\gamma$ where $\alpha$ is the intercept, $\beta$ the slope and $\gamma$ the asymptote. Each condition was fit individually; Table 1 gives the mean parameters and confidence intervals (Fig. 3B presents a fit on data pooled across participants for illustrative purposes). None of the parameters differed between conditions, as evidenced by the overlapping confidence intervals. In all conditions, the asymptote was about 1 and the intercept not different from 0. Although the slope was descriptively shallower in the same-cluster condition, the difference was not statistically significant.

**Table 1:** Parameter fits for the exponential relationship between serial dependence ratio and trials in the past, for each of the four trial pair conditions.

	$\alpha$ (intercept)	$\beta$ (slope)	$\gamma$ (asymptote)
Same cluster	−0.41 [−0.93; 0.12]	−0.10 [−0.26; −0.06]	1.04 [1.02; 1.08]
Orthogonal cluster	−0.47 [−1.07; 0.26]	−0.34 [−0.63; −0.05]	1.00 [0.96; 1.04]
Nearby on axis being rated, far on other axis	−0.28 [−0.88; 0.45]	−0.19 [−0.43; −0.03]	0.99 [0.94; 1.03]
Far on axis being rated, nearby on other axis	−0.55 [−0.98; 0.10]	−0.29 [−0.52; −0.13]	1.04 [1.01; 1.09]

### Stereotype content leads to reproducible neural patterns

**Figure 4:**
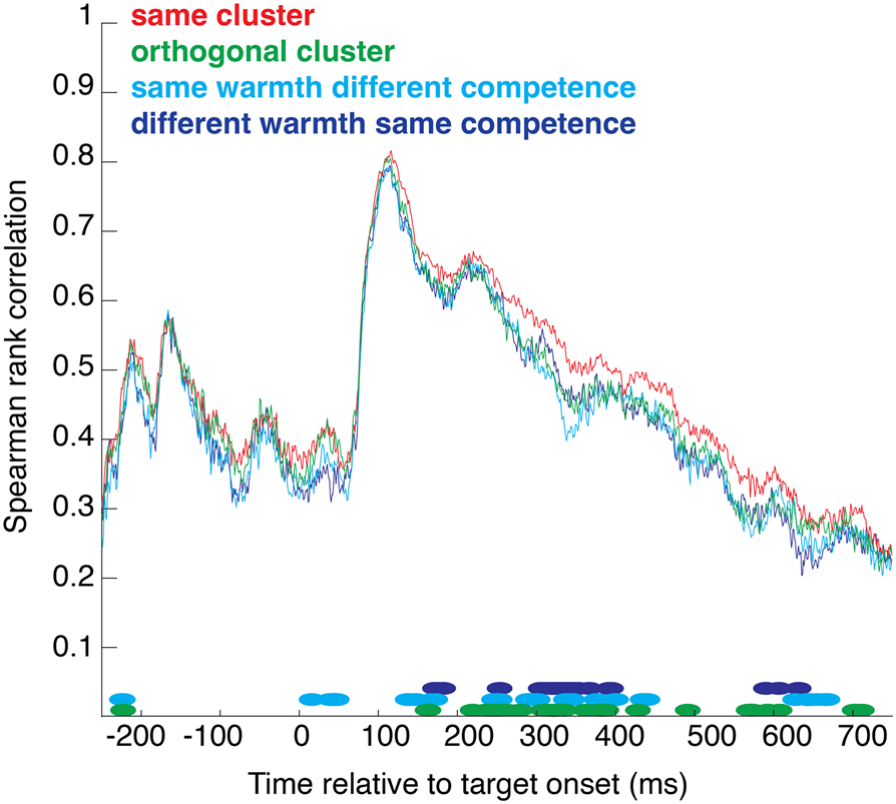
Representational similarity analysis. Spearman rank correlation between subsets of trials from the same cluster (red), orthogonal clusters (green), clusters with same warmth different competence (light blue) or clusters with different warmth same competence (dark blue), time-locked to stimulus onset. Marks at the bottom of the graph indicate time points that differed significantly from the same-cluster condition.

We used representational similarity analysis to examine the neural patterns evoked by stereotype content. Figure 4 presents the bootstrapped correlation between subsets of trials from the same cluster, versus the correlation between a subset of trials from one cluster and a subset of trials from the orthogonal cluster, that share no representational content—neither warmth nor competence. Greater similarity in the same-cluster condition suggests the particular content of a given stereotype (e.g. high-warmth-high-competence) is represented by a particular neural pattern. The difference emerged at ~ 200 ms and stayed significant for several time points up to ~ 600 post-stimulus (significance was ascertained by the false discovery rate procedure as described by^37^).

We also compared the brain response to a given cluster with that evoked by a cluster that differed on competence but not warmth, or on warmth but not competence. Light and dark blue curves in Fig. 4 correspond to these two conditions. They both line up rather nicely with the orthogonal cluster, and this was confirmed by the permutation test which revealed similar significant differences with the same-cluster condition.

**Figure 5:**
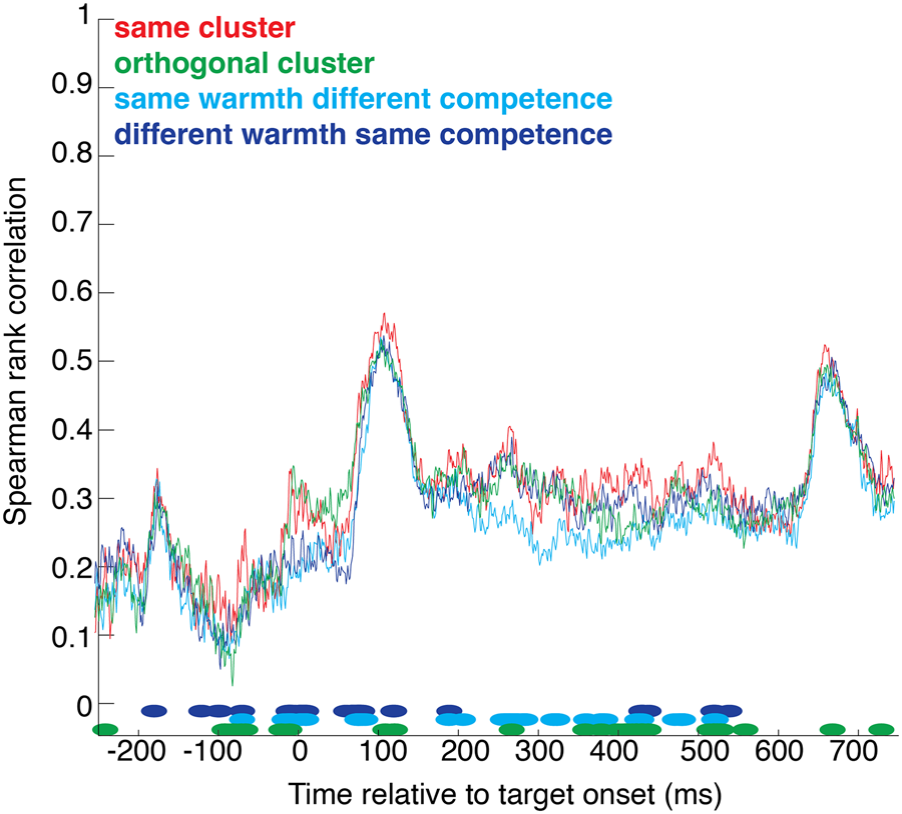
Representational similarity analysis. Spearman rank correlation between subsets of trials preceded by a trial from the same cluster (red), from an orthogonal cluster (green), from a cluster with same warmth different competence (light blue) or from a cluster with different warmth same competence (dark blue), time-locked to stimulus onset. Marks at the bottom of the graph indicate time points that differed significantly from the same-cluster condition.

We also examined the extent to which the neural response to a given social group was influenced by the past—the neural correlate of SD found in behavior. Figure 5 shows the correlation between subsets of trials preceded by a trial from the same cluster, versus the correlation between a subset of trials preceded by a same-cluster trial and a subset of trials preceded by an orthogonal-cluster trial, or versus the correlation between a subset of trials preceded by a same-cluster trial and a subset of trials preceded by a trial sharing warmth (or competence) content, but not competence (or warmth).

Significant differences were visible before target onset and stayed significant for several time points up to ~ 600 ms post-stimulus. The significant differences before target onset suggest that there was a pre-existing state or set onto which the current response builds.

## Discussion

Ratings of social groups on warmth and competence were serially dependent. Behavioral performance revealed a pattern of results showing two hallmarks of SD. First, the attractive effect decreased with time, reminiscent of the temporal tuning found with visual stimuli. Second, SD between social groups was feature tuned: attraction between successive trials occurred only between groups that were nearby in the warmth and competence space. Results were compatible with the psychological non-separability of the two dimensions. Indeed, SD occurred strongly when two successive groups were nearby in feature space, and that “nearby” had to be on both dimensions. When the two successive groups were nearby on one axis but far on the other, there was less SD, showing that the two dimensions of warmth and competence are not separable.

Another hallmark of SD is its spatial tuning. That was impossible to test here, as all stimuli were presented at screen center. Furthermore, it is unclear whether spatial tuning would have any meaning in the context of non-visual, non-spatial stimuli such as social groups.

EEG results suggest that a given stereotype content, characterized by warmth and competence ratings, evokes a reproducible brain pattern. Stereotypes that differ on any axis (warmth, competence or both) lead to an identifiably distinct patterns of activation. Sharing similar content on one of the representational axes (warmth or competence), but not the other, is as different as not sharing any representational content. This result is coherent with the behavioral data suggesting that the two axes are not psychologically separable; the axes are not neurally separable, either.

Our study complements previous research showing that social information and representations can be tracked with brain imaging (for a review:^38^). For example, Chavez & Heatherton (ref.^39^) performed a representational similarity analysis of fMRI data recorded while participants viewed photos varying in sociability from positive (e.g. friends laughing) to negative (e.g. depictions of pain). The authors found a high degree of similarity within social dimensions in a ventral portion of the medial pre-frontal cortex. The neural similarity structure was correlated with a behaviorally measured similarity structure of the same stimuli, suggesting that a psychologically meaningful representation of social information is reflected in the neural activity. This is reminiscent of our finding of shared neural patterns between social groups of similar warmth and competence, and concurrent behavioral interactions (in the form of serial dependence) between these same groups.

Parkinson, Liu & Wheatley (ref.^40^), again using representational similarity analysis of fMRI data, found a metric for distance that was common between spatial (near versus far), temporal (recent versus long ago) and social (close friend versus distant acquaintance) stimuli. In other words, a distinct pattern of neural activity differentiates socially close from socially distant individuals, and this distinction was similar to that found between near versus far objects and points in time. This is reminiscent of our finding of a distinct neural pattern differentiating social groups judged as low versus high warmth, and low versus high competence.

Serial dependence has been described as the result of a persistence of visual working memory traces^35,41–43^. If one extends this account to include all working memory traces, not only visual, then this may explain SD in all sorts of tasks, including rating social groups. When faced with an individual who belongs to a social group, the representation of that group may become activated in working memory, and remain active there for a few seconds, thus potentially interacting with subsequent representations. This interpretation is supported by our observation that the temporal decay (or temporal tuning) of social group representations in working memory is comparable to low-level visual representations^44^.

Our findings on the neural correlates of serial dependence align with some previous work. For example, using multivariate pattern analysis (MVPA), several authors have shown that it is possible to train a classifier (e.g. a support vector machine) to distinguish patterns of brain activity evoked by objects preceded by object A versus those preceded by object B, and then successfully categorized novel trials as a function of the preceding object^45,46^. These results, like the present results, show that the current trial contains information about the previous trial. Luo & Collins (ref.^47^) examined the EEG brain response of observers to prototypical objects (faces, cars, and houses) and morphs that mixed properties of two prototypes. Behavior was biased toward previously seen objects. Representational similarity analysis revealed that responses evoked by visual objects contained information about the previous stimulus. Similarly to the results we found in the current study (see the significant data points to the left of zero in Fig. 5), the influence of the past trial on the current trial occurred even before target onset, suggesting that there was a pre-existing state or set onto which the current response built. Other studies, however, have suggested that decodable traces of prior stimuli are first repulsive and then attractive^48,49^. This may be related to reports of behavioral repulsion from recent stimuli (e.g.^50–52^). Indeed, history effects grouped under the term “serial dependence” are sometimes attractive, as in the current study, and other times repulsive. A comprehensive theory of which conditions influence the direction of the effect has yet to be proposed. Taken together, what is known about the neural correlates of SD is still somewhat mixed and further research is needed.

## Methods

The following sections describe the two surveys that we used to identify social groups and quantify their perceived warmth and competence in and by French society. They were run prior to the pre-registration of the main experiment^53^, anonymously and online.

### Survey 1: finding the groups

The goal of this short survey was to identify the social group labels for use in the long survey. Volunteers were recruited through the Paris Cité University community. Age and gender were recorded, but no other identifying information. The survey took 3–5 min to complete. We aimed to match the sample sizes from Fiske et al. (ref.^2^), n = 31, and Cuddy et al. (ref.^16^), n = 30/53/28 (3 experiments). 55 respondents completed the survey: 41 women (75%), mean age 32 years old, age range 18–72. We selected the groups cited by at least 5 respondents, for a total of 32 groups. These groups were: femmes, riches, hommes, pauvres, cadres, classe moyenne, LGBT, personnes âgées, jeunes, noirs, arabes, chômeurs, hétérosexuels, ouvriers, immigrés, étrangers, blancs, enfants, retraités, étudiants, asiatiques, handicapés, homosexuels, SDF, travailleurs, croyants, personnes de droite, fonctionnaires, personnes de gauche, non-binaires, religieux, campagnards [women, rich, men, poor, executives, middle class, lgbt, elderly, young, black, Arab, unemployed, heterosexual, workers, immigrants, foreigners, white, children, retired, students, Asian, handicapped, homosexual, homeless, workers, believers, right-wing, civil servants, left-wing, non-binary, religious, rural]. In the final sample of groups for the long survey, we also included seven groups taken from Carrier, Louvet & Rohmer (ref.^54^) and four groups from other studies to increase the likelihood that we would find groups clearly differentiating high and low competence: manœuvres de chantier, éboueurs, vendeuses, chefs d’entreprise, ingénieurs informatiques, infirmières, avocates, diplômés de l’enseignement supérieur, comptables, femmes au foyer, féministes [construction workers, sweepers, saleswomen, company directors, computer engineers, nurses, lawyers, educated, accountants, housewives, feminists]. This yielded a total of 43 groups.

### Survey 2: quantifying warmth and competence for each of the social groups

The 43 groups identified in the short survey were presented to a new set of 167 volunteers, recruited through the University Paul-Valéry Montpellier 3 student community. 151 were women (90%), 14 men (8%), and 2 preferred not to respond (1%). Mean age was 21 years old and ranged from 17 to 49. Again, sample size was aimed to at least match benchmarks from the two studies mentioned above (Fiske et al., 2002, n = 74; Cuddy et al., 2009, n = 60/82/91). For each group, participants had to respond to one question about warmth, selected from the following set:

Du point de vue de la société, dans quelle mesure les membres de ce groupe sont-ils perçus comme… [To what extent does society view the members of this group as …]

- étant amicaux ? [friendly?]
- étant chaleureux ? [… warm?]
- ayant un bon fond ? [… good-natured?]
- étant sincères ? [… sincere?]

For each group, participants had to respond to one question about competence, selected from the following set:

Du point de vue de la société, dans quelle mesure les membres de ce groupe sont-ils perçus comme étant… [To what extent does society view the members of this group as …]

- compétents ? [… competent?]
- confiants ? [… confident?]
- capables ? [… capable?]
- habiles ? [… skillful?]

Participants gave their responses on a scale from 1 (not at all) to 7 (completely). The entire survey took approximately 20 min to complete.

**Figure 6:**
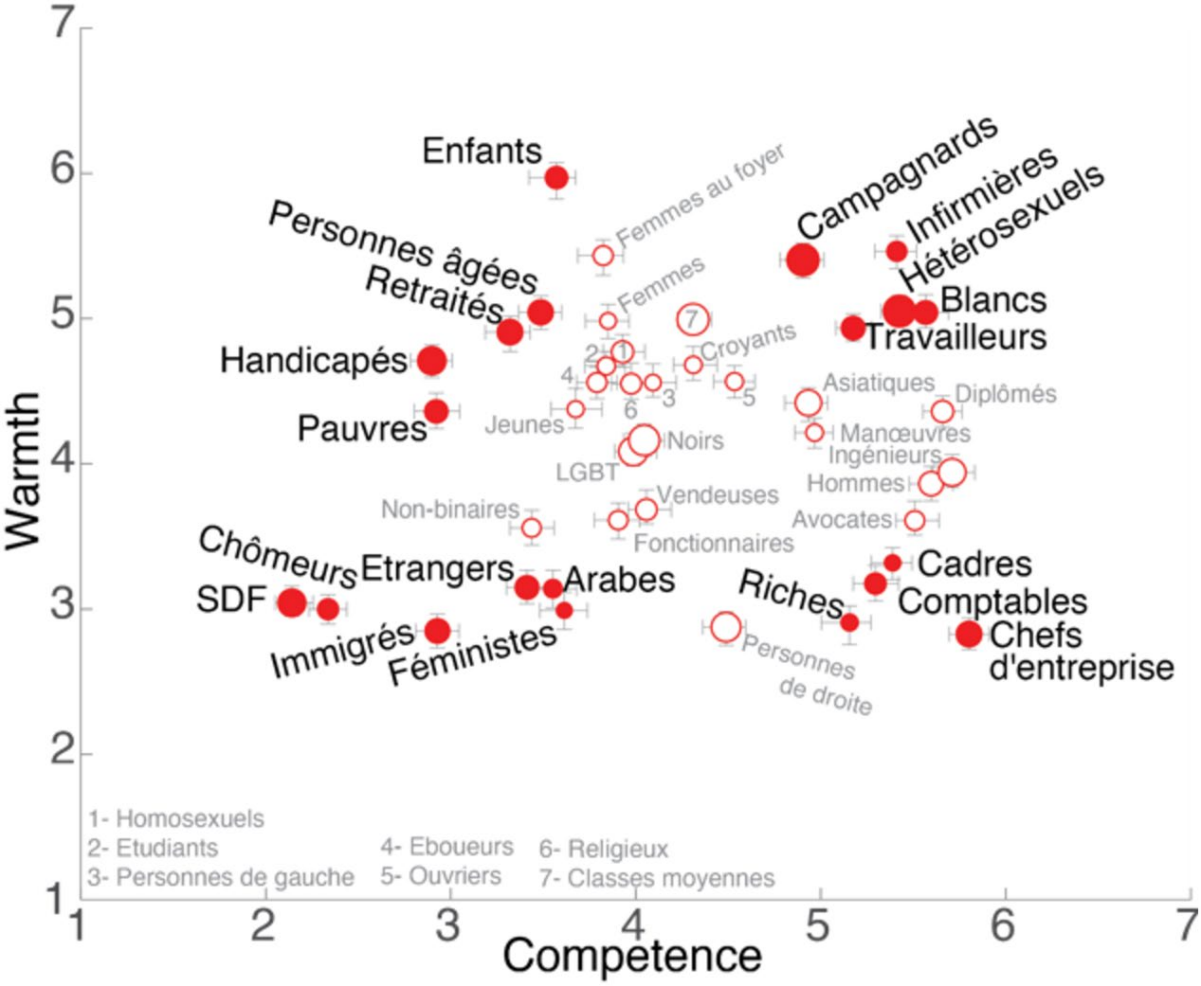
Warmth and competence scores obtained for each group in the long survey. Data points represent the mean; their size, the total number of ratings given (minimum: 119, maximum: 147) and error bars, standard error of the mean. Filled data points are the 20 groups selected for the main experiment.

Figure 6 presents the mean scores for each group on warmth and competence scales.

We aimed to identify five clusters of groups. First, the four corners, i.e. with high or low values on one or both scales. Second, a cluster with intermediate scores on both axes which we eliminated (represented in Fig. 6 as open red symbols and smaller, gray font.) The final sample of social groups to be included in the main experiment was composed of the following 20 groups: riches, pauvres, cadres, personnes âgées, arabes, chômeurs, hétérosexuels, immigrés, étrangers, blancs, enfants, retraités, handicapés, SDF, travailleurs, campagnards, chefs d’entreprise, infirmières, comptables, féministes [rich, poor, executives, elderly, Arab, unemployed, heterosexual, immigrants, foreigners, white, children, retired, handicapped, homeless, workers, rural, company directors, nurses, accountants, feminists].

## Main experiment

### Subjects

36 subjects, mostly right-handed, participated in the experiment, including 23 women and 13 men, aged from 18 to 45 years old (mean = 29.1 ± 8.2 years old). They were recruited from the INCC laboratory community and mostly from the subject pool maintained by the Relais d’Information sur les Sciences de la Cognition (UMS ENS-CNRS 3332), a service maintained by the Centre National de Recherche Scientifique. All were native French speakers and had normal uncorrected vision and self-reported no psychological or neurological deficits. After giving informed consent, they were set up with the EEG recording system. At the end of the experiment, they were compensated with gift vouchers (15 €/h). The study was approved by the local ethics committee (CPP Ouest IV-Nantes, protocole 52/20_3). All research was performed in accordance with relevant guidelines/regulations and the Declaration of Helsinki.

We estimated the number of participants in the following manner. Because there have been no previous investigations of serial dependence of social groups, to our knowledge, we looked at effect size and power across several different kinds of stimuli in the following publications:

- Fischer & Whitney, 2014 (ref.^2^), main experiment (Gabor patches), number of participants necessary to match their effect size, n = 3;
- Collins, 2019 (ref.^55^), Experiment 1 (Gabor patches), n = 3;
- Collins, 2019, Experiment 2 (Gabor patches), n = 4;
- Collins, 2019, Experiment 3 (Gabor patches), n = 3;
- Collins, 2022 (ref.^29^), Experiment 1b (shapes), n = 7;
- Collins, 2022, Experiment 2 (facial emotional expression), n = 4;
- Liberman, Fischer & Whitney, 2014 (ref^30^), Experiment 1A (facial identity), n = 7;
- Liberman, Fischer & Whitney, 2014, Experiment 1B (facial identity), n = 7.

We reasoned that the complexity of the social stimuli that participants had to rate may lead to smaller SD effects than those reported with visual stimuli, and also because we planned to use EEG, we aimed to collect data from a higher number of subjects because this was feasible given the time constraints on data acquisition. A data collection period was thus defined over the course of eleven weeks from February 10th to April 30th, 2023.

### Apparatus

Stimuli, designed with MATLAB and Psychtoolbox^56^, were back‐projected by a ProPixx projector (VPixx Technologies, Saint‐Bruno, Québec, Canada) onto a wide screen (107 × 60 degrees of visual angle, dva) with a resolution of 1920 × 1080 pixels and a refresh rate of 120 Hz. Participants sat about 1 m from the screen in an otherwise dark room. Viewing was binocular. Movements of the dominant eye were monitored with an Eyelink 1k Plus (SR Research, Mississauga, Ontario, Canada) at 1000 Hz sampling rate. At the beginning of a session, the Eyelink was calibrated with the standard 5‐point Eyelink procedure. Before each trial, fixation was checked for 200 ms and if the measured value was greater than 3 dva, a new calibration was initiated. Calibration was also automatically renewed every 200 trials. Participants were required to maintain their gaze within a tolerance window of 4 dva from the center of the visual images. If their gaze deviated from this window, the trial was aborted and rerun right after a warning was shown to encourage fixation.

### Stimuli & procedure

A red fixation dot (diameter 0.5 dva) was presented at screen center. Stimuli were composed of a series of black-on-white silhouettes and 20 social group names. Silhouettes and names were presented simultaneously at screen center for 750 ms, followed by a response screen (Fig. 1). The response screen was composed of an adjective (the same as those used in the long survey, see above) and a bar that appeared gradually within 1000 ms below the stimulus. Participants were to move the mouse pointer, which always appeared slightly below the response bar center, and click to indicate the extent to which the person corresponded or not to the adjective. Adjectives were either related to warmth or competence, and the probability of switching between the two scales was 20%. Other than that, the social group name, adjective, and silhouette were randomly determined. We included silhouettes to introduce some variability in responses; otherwise, participants would have been asked to rate the same group on the same adjective multiple times, which appeared to us to lack in experimental (or situational) realism, and thus to pose a threat to external validity. Thus, the question we asked was “to what extent is this person, who is a member of group [x], seen by French society as [adjective]”.

**Figure 7:**
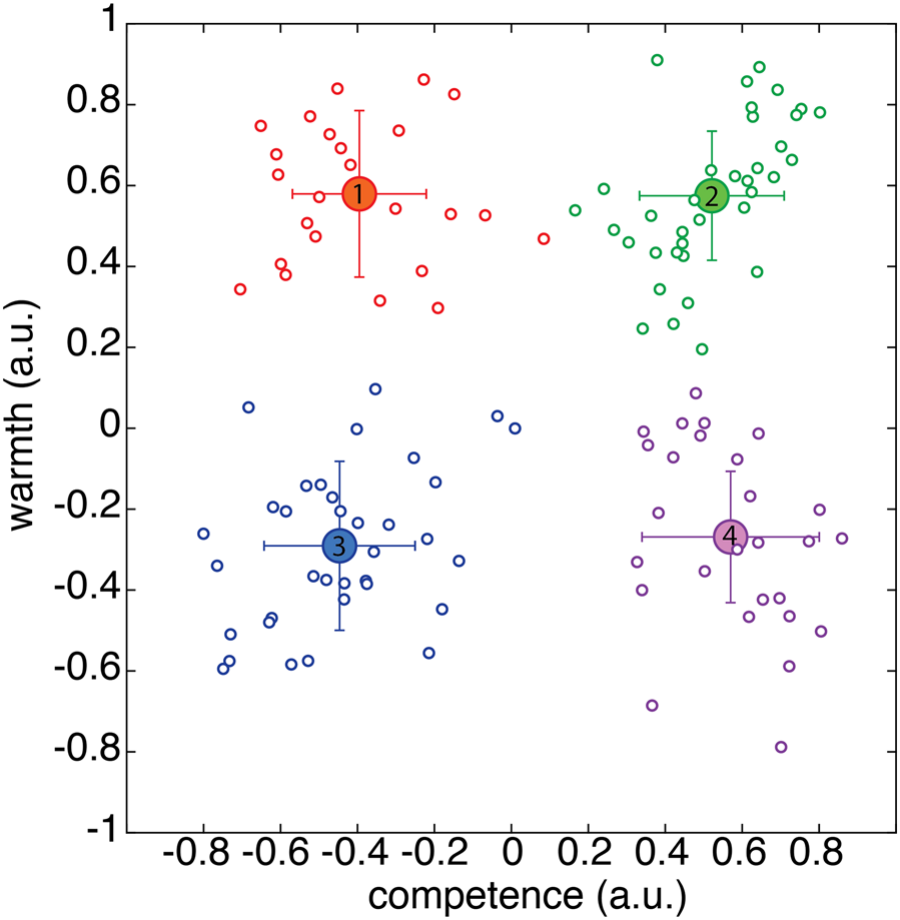
Average ratings, on each axis, pooled across all participants, for each of the four clusters: 1—low-competence-high-warmth, 2—high-competence-high-warmth, 3—low-competence-low-warmth, 4—high-competence-low-warmth. Large filled data points with error bars correspond to the average and standard deviations; small open data points to the individual observers. Ratings are given in arbitrary units from − 1 to + 1 (the extent of the response bar).

Although Survey 2 aimed to identify social groups that led to relatively constant ratings from our population of mostly French university students, there was some variability between observers. While most people evaluated feminists as high competence but low warmth, some participants evaluated feminists as both warm and competent. If we uniformly considered this group as part of the low-warmth-high-competence cluster, we may introduce quite some noise into our measures. Instead, we identified the groups that made up the four clusters individually, such that all groups in observer 1’s high-warmth-high-competence cluster did not necessarily match the groups in observer 2’s high-warmth-high-competence cluster. Some observers gave central ratings to some social groups that could therefore not readily be attributed to a particular cluster. In this case, that group was removed from further analysis. Furthermore, some observers used the entire rating scale, while others tended to give more central ratings. Figure 7 shows both average and individual ratings.

### Data analyses

We measured serial dependence by looking at response error as a function of relative distance between successive trials. To do so, we calculated an error term for each trial: the difference between the score given on a particular trial and the average score for that group, measured across the entire experiment (each group was presented approximately 20 times). Error was then related to the relative difference between two successive trials, defined as the difference between the average scores of groups presented on two successive trials. The DoG was given by $error=x\alpha wc{e}^{{-\left(wx\right)}^{2}}$ where $x$ is the relative warmth or competence of the previous trial, $\alpha$ is the amplitude of the DoG curve multiplied by the constant $c=\frac{\sqrt{2}}{{e}^{-0.5}}$ which scales the amplitude to the curve peak in y units (e.g., units on the rating scale), and $w$ is the inverse of the curve width. Data was first pooled across participants and then fit.

Representational similarity analysis (RSA) was applied to the EEG data. RSA quantifies the correlation between the spatial patterns of activity evoked in different experimental conditions (^57^). Here, patterns correspond to the voltage recorded at each electrode (n = 127) and at each time point. For each participant and each time point from 250 ms before stimulus onset to 750 ms after stimulus onset, we computed Spearman rank correlations between patterns on a random subset of half of the trials for each pair of conditions. For example, we split the same-cluster condition into two random halves and correlated the two subsets. For the orthogonal cluster comparison, we took a subset of trials from one cluster and a subset of trials from the orthogonal cluster, that share no representational content—neither warmth nor competence. This same procedure was applied to compared the brain response to a given cluster with that evoked by a cluster that differed on competence but not warmth, or on warmth but not competence. For all of these comparisons, we repeated the procedure ten times, each time taking a different random split of trials. Significance was ascertained with the false discovery rate (FDR) procedure (Storey^37^); significant time points are identified by points in Figs. 4 and 5.

### EEG pre-processing

Electroencephalographic activity was recorded with 128 Ag/AgCl electrodes mounted on a fabric cap and amplified by an ActiCHamp Plus amplifier (Brain Products) at a sampling rate of 1000 Hz. Electrodes were arranged according to the international 10–20 system, with the left and right mastoids as online references. Preprocessing of the EEG data was performed with the Matlab-based FieldTrip Toolbox^58^. The following processing steps were applied: high-pass filtering at 0.75 Hz and stop-band at 50 Hz with deletion of the linear component, performed on the continuous signal; visual detection of electrodes with poor signal (average of 2 ± 2 electrodes per participant) and reconstruction by interpolation of neighboring electrodes; epoching around the onsets of the image, from − 250 ms to + 750 ms (no epochs were removed); re-referencing to the average of the electrodes; baseline correction over the 250 ms preceding the onset of the stimulus.

## Data Availability

Data is available from the corresponding author on reasonable request.
